# Accumulation and risk assessment of metals in palm oil cultivated on contaminated oil palm plantation soils

**DOI:** 10.1016/j.toxrep.2020.01.016

**Published:** 2020-01-27

**Authors:** O.B. Olafisoye, O.S. Fatoki, O.O. Oguntibeju, O.A. Osibote

**Affiliations:** a Faculty of Applied Sciences, Department of Chemistry, Cape Peninsula University of Technology, P.O. Box 652, Cape Town, 8000, South Africa; b Faculty of Health and Wellness Sciences, Department of Biomedical Sciences, Cape Peninsula University of Technology, P.O. Box 1906, Bellville, 7535, South Africa; c Faculty of Applied Sciences, Department of Mathematics and Physics, Cape Peninsula University of Technology, P.O. Box 652, Cape Town, 8000, South Africa

**Keywords:** Metals, Oil palm plantations, Palm oil, Soil, Micro emulsion, ICP-OES, Health risk assessment

## Abstract

Anthropogenically polluted soils with metals are detrimental to human life. The present study assessed the concentration of metals in soil and the risks associated with the consumption of the metals when transferred from soil to palm oil. The metals of interest were Cd, Co, Cr, Cu, Fe, Mn, Ni and Zn. Analysis was done on metals in soil and virgin palm oil from fifteen independent sampling locations in the southern states of Nigeria, which includes the Nigeria Institute for Oil Palm Research (NIFOR) and its substations. Top soils were collected at a depth of 0−15 cm and virgin palm oil in 1 litre container by grab sampling method. The method proposed was to achieve high throughput with minimal mobile phase solvent. Micro emulsion technique was involved as sample preparation method for the extraction of metals in virgin palm oil from the matrix. Inductively Coupled Plasma Optical Emission Spectroscopy (ICP-OES) was used to analyse metals in virgin palm oil and soil. This reduces analysis time and does not require complex sample preparation. Zinc metal recorded the highest concentration of metal in the palm oil and Chromium metal recorded the highest concentration of metal in soil. There was a correlation between the accumulations of metals from soil to palm oil. Risk was assessed using various indices. Cadmium metal recorded the highest concentration in the Target Hazard Quotient (THQ), Accumulation Factor (AF) and Health Risk Index (HRI). Daily Intake of Metals (DIM) was highest for Chromium metal. Cadmium was the highest accumulator of metals in the palm oil.

## Highlights

• Risk assessment translocation of metals from soil to palm oil was studied.
• Metal sequence -palm oil/soil: Cd>Fe>Co>Ni>Zn>Cr>Mn >Cu / Zn>Cr>Cu>Ni>Fe>Mn>Co >Cd.
• Risk transfer from soil to palm fruit follow the trend Cd > Fe > Co > Ni > Zn > Cr > Mn > Cu.
• Daily Intake of Metals, Health Risk Index, Accumulation Factor values above unity.

## Graphical abstract

**Figure:**
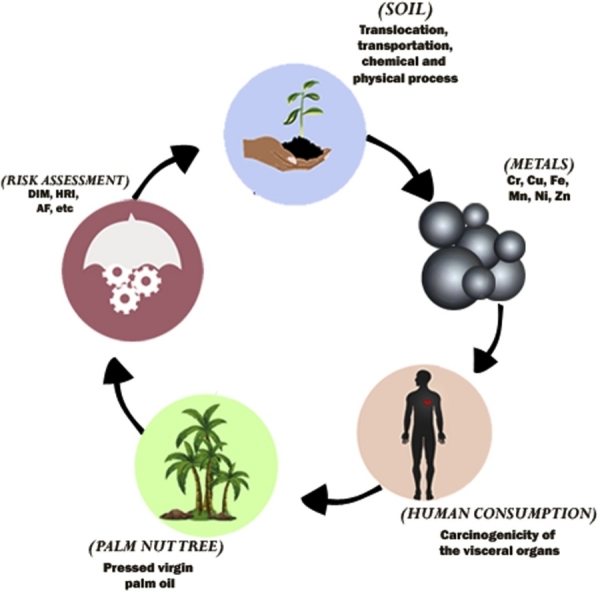

## 1 Introduction

A major pathway through which metals enter soils is by anthropogenic means [1,2]. Plant species significantly accumulate metals in various parts especially the fruits thereby remediating soils. This leads to bioaccumulation and bio magnifications in the food chain and the disruption of chemical and biological activities in the human body [3,4]. The routes through which metals enter the food chain are via inhalation and ingestion. Although literature reveals that, some species of plants can bio accumulate metals more than the other species [5]. Anthropogenic sources of metal pollution are mostly from industries and unwholesome farming practices. Wastewaters from factories usually drain to farmlands elevating the levels of metals in soils. The populace is incessantly exposed to metals through the consumption of food grown on such soils. Numerous literatures supported this fact [[6], [7], [8], [9], [10]].

The study of metals in soils requires systematic assessment as monitoring approach to eradicate the effects on the food chain. Risk assessment have been studied using various tools such as the Accumulation Factor (AF), Hazard Quotient (HQ), Health Risk Index (HRI), Daily Intake of Metals (DIM), Target Hazard Quotient (THQ), Morbidity Status (MS), Enrichment Factor (EF), and Degree of Contamination (C_deg_) [2]. Overtime, non-essential metals bio accumulates in plant parts that become toxic to animals and humans when consumed as food. Application of fertilizers, untreated sewage water from municipal and industrial applications, leaching from dumpsites, fertilizer application, and waste from mining have contributed significantly to the build-up of metals in soils.

The oil palm tree (*Elaeis guineensis)* is a perennial crop. It is an excellent source of fatty acids, minerals, and vitamins for the food and chemical industry [11]. Palm oil from the oil palm fruit has been proven to cure various diseases especially cancer and heart maladies [[12], [13], [14], [15], [16]]. Nigeria is a tropical country characterized by a wet and dry climate at various times of the year. This is particularly beneficial for the production of the oil palm fruit. The oil palm fruit produces palm oil that is a stable ingredient in most local dishes consumed by Nigerians [17]. Metals are also present in palm oil due to various reasons ranging from environmental and processing operations to unwholesome agronomical methods. Ayodele and Oluyomi in 2011 [9] reported on the issue of contamination arising during cultivation of the oil palm tree and harvesting of the oil palm fruit due to pre-planting and post-planting farming practices such as the use of fungicides and fertilizers residues. Contamination may also arise from a number of factors that includes washing of the oil palm fruit with water from lead/metal pipes or rivers. In addition, mechanical stripping of the oily flesh from the oil palm fruit, cooking and sieving of the flesh to obtain the virgin palm oil, corrosion of the processing equipment and storage in large metal vessels; are sources of metal contamination [18]. Other sources of anthropogenic contamination may also arise from the oil palm plantation situated close to domestic and industrial sources of pollution [8].

The determination of elements in virgin palm oil is of economic importance because palm oil is a very important cash crop for the food and chemical industry. It is essential to determine the presence of metals in palm oil as this is one of the criteria for oil quality. Metals in palm oil are deleterious because this affects the shelf life and aesthetic quality of the oil. Metals in the oil enhance the oxidation of fatty acids to esters, which affects the nutritional value and the properties of the oil [[19], [20], [21]]. Metals are toxic at elevated concentrations. However, some metals are essential to humans, their presence in palm oil, which is a raw material for a variety of food and chemical industries may not be ideal for production processes. This is particularly an issue in the production of biodiesel due to the presence of such metals that consequently affects the quality of the oil.

Hence, an analysis was performed to determine metals (Cadmium Cd, Cobalt Co, Chromium Cr, Copper Cu, Iron Fe, Manganese Mn, Nickel Ni, and Zinc Zn) in virgin palm oil from fifteen independent sampling locations. The proposed method of micro emulsion as sample preparation method alongside with the instrumental analysis using the ICP-OES for this analysis was initiated to achieve high throughput and low flow rate with minimal mobile phase solvent. It was able to eradicate the impending problem of former analysis that was majorly the dense matrix of oil samples and the concentration of metals in oil samples at very critical concentrations [22]. Analytical chemists have also employed several instrumental techniques for the determination of elements in oil samples, the most common technique being Inductively Coupled Plasma Optical Emission Spectroscopy (ICP-OES) [9,8,23]. The determination of elements in oil using AAS recently by some scientists was not adequate due to the nature of the matrix and the difficulty in separating the analyte from such matrix [24,25]. This could be attributed to the instability of the analyte in the oil and the high organic content of the oil. The use of hazardous solvents has to be employed to destroy or dissolve the matrix as the case may be so that the analyte may be free in solution. Sample separation and pre-treatment steps were long and laborious. Such sample preparation steps such as solid phase extraction, microwave assisted acid digestion and wet/dry ashing, involving hazardous acids or a mixture of both was normally employed. Another disadvantage of using AAS process was the use of very expensive organometallic standards for instrument calibrations. The proposed method eradicated the use of hazardous chemicals and the use of expensive unstable organometallic standards for calibration [25,26]. Hence, micro emulsions were employed as sample preparation method for the determination of metallic elements in this study. The micro emulsions were stable thermodynamically when tested using a UV spectrometer after being observed for about three hours. The micro emulsions were composed of water, oil, and alcohol. In addition, the micro emulsion particles were homogenous and were actually in the order of 5−100 nm in size suspended in a phase that is continuous [[27], [28], [29]].

The proposed ICP-OES analysis using oil-water micro emulsion as sample preparation method was not only reproducible, accurate and reliable but also equally convenient because it provided a sample extraction capable of breaking down the complex matrix in virgin palm oil. This was for metal analysis by ICP-OES [30].

## 2 Materials and methods

### 2.1 Sampling and chemicals

**Fig. 1:**
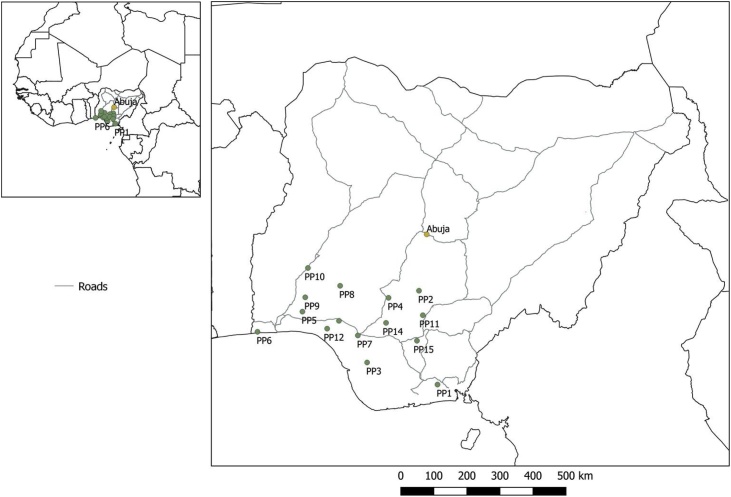
Map of Nigeria showing the sampling locations.

Virgin palm oil and soils were sampled from fifteen sampling locations in the Nigeria Institute for Oil Palm Research (NIFOR); the headquarters in Benin city and substations in the south western area of Nigeria. The choice of the sampling areas was based on Oil Palm Plantations in the region receiving significant input of pollution characteristics from crude oil in crude oil producing areas of the region. Other inputs were from effluents of domestic, industrial, and electronic waste. Oil palm fruits were pressed from the bunch and the virgin oil was collected in 1 l plastic containers in all the sub sites by random sampling for each site. The collected palm oil samples were mixed in order to achieve a grab sample and to give a true representative of all the plantations. Choice of sampling sites was also based on densely populated areas. Soil sampling was also performed by random and grabs sampling. The soils were collected from a depth of 0−15 cm. Majorly the choice of the sampling areas were based on oil palm plantations in the region receiving significant input of pollution characteristics from crude oil in crude oil producing areas of the region. Other inputs were from effluents of domestic, industrial, and electronic waste. Soil samples were collected from topsoil using a spiral auger of 2.5 cm at a depth of 0−15 cm. A total of fifteen soil and virgin palm oil samples, one from each site was transferred into cleaned polythene bags and 1-litre containers for soil and virgin palm oil samples respectively, labelled and transported to the laboratory. Fig. 1 shows the sampling locations. They were stored in pre sterilized ice chest at lower temperatures prior to transportation to the laboratory for chemical analysis. The soil samples were air dried for over a week, ground with a porcelain mortar and pestle, passed through a 2 mm aperture sieve, further dried in the oven at 105 ± 5 °C for about 4 h, well labelled accordingly, and kept in clean polythene bags for further analysis. The virgin palm oil samples were kept in a cool dry cupboard awaiting further analysis. All apparatus used for the analysis have been previously soaked in nitric acid, washed with detergent, and rinsed twice with deionized water. The sampling containers were sterilized and the samples preserved in a refrigerator prior to analysis in the laboratory. High purity analytical grade chemicals and reagents (purchased from Sigma Aldrich (USA) and Merck, (Germany.) were used alongside deionized water (Milli-Q water, Belford, America). Standard calibration curves for the analysis were prepared from single element standards that were certified and traceable to the National Institute of Standards and Technology (NIST). Non-serial dilutions of much lower concentrations of the standards were also prepared in making the calibration curve throughout the analysis.

### 2.2 Analysis of soil samples

The soil samples were tested for parameters such as the electrical conductivity, pH and organic matter. The pH of the soil was measured with a pH meter in a suspension of the soil and water [31]. One part of soil by weight to 5.0 parts of deionized water by volume was measured and analysed using the glass electrode pH meter. The solid to liquid ratio of 1:5 soil: deionized water suspension is sensitive to seasonal variation in the pH of soil solution. Organic matter was measured using the modified Walkey and Black method [32]. Total metal extraction was accomplished using the microwave assisted digestion method.

### 2.3 Analysis of palm oil samples by the micro emulsion method

About 0.5 g of each of the oil samples was accurately weighed into a 100 mL polypropylene tube with lid. HCl was added (c.a 2 mL) and propan-2-ol to a final volume of 10 mL. The mixture was shaken vigorously on a shaker for 30 mins to obtain an evenly dispersed homogenous emulsion that remained stable without dispersing when left for about four hours [33,34]. This was kept in the fridge at 4 ℃ for metallic element analysis using the ICP-OES. The instrument of choice for the analysis of metals in soil and the palm oil was the Inductively Coupled Plasma Optical Emission Spectrometer (ICP-OES) [23,25,35]. In order to validate the analytical method, the following method validation parameters such as instrumental detection limit, limit of detection, limit of quantification, precision, and accuracy studies were carried out. The Instrumental detection limit (IDL) is the smallest signal above background noise that an instrument can reliably detect. This can be calculated to be the concentration that is equal to thrice the standard deviation of the signal produced by the blank. In this study, the IDL for each metal was calculated from the analysis of seven replicates of the blank concentration. This was represented as; IDL = 3 X Sbl; where Sbl is the standard deviation of the calibrated blank solution.

The limit of detection (LOD) is the minimum concentration of the analyte that can normally be detected without necessarily quantifying it. This has an acceptable level of uncertainty. Calculations for the LOD for each metal was made by determining the analysis of seven replicates of the method blank which was digested in the same way as the samples. This was represented as; LOD = 3 X Sbl; where Sbl is the standard deviation of the method blank solution.

The limit of quantification (LOQ) is the lowest concentration of an analyte in the sample which can be quantitatively determined with some levels of uncertainty. This can be achieved in triplicate of seven method blanks which are digested as the actual samples. This is represented as; LOQ = 10 X Sbl; where Sbl is the standard deviation of the method blank solution [36,37].

### 2.4 Accumulation factor calculation (AF)

The movement of metals from the soil to various parts of the plants especially the edible parts can be described by the accumulation factor using Eq (1) [2,38]

(1) $$\text{A}\text{F}=\frac{\;\text{H}\text{e}\text{a}\text{v}\text{y}\;\text{m}\text{e}\text{t}\text{a}\text{l}\;\text{c}\text{o}\text{n}\text{c}\text{e}\text{n}\text{t}\text{r}\text{a}\text{t}\text{i}\text{o}\text{n}\;\text{i}\text{n}\;\text{v}\text{i}\text{r}\text{g}\text{i}\text{n}\;\text{p}\text{a}\text{l}\text{m}\;\text{o}\text{i}\text{l}}{\text{H}\text{e}\text{a}\text{v}\text{y}\;\text{m}\text{e}\text{t}\text{a}\text{l}\;\text{c}\text{o}\text{n}\text{c}\text{e}\text{n}\text{t}\text{r}\text{a}\text{t}\text{i}\text{o}\text{n}\;\text{i}\text{n}\;\text{t}\text{h}\text{e}\;\text{s}\text{o}\text{i}\text{l}}$$

### 2.5 Metals daily intake (DIM)

Eq. (2) was used in the calculation of the daily intake of metals ingested by human [39,40].

(2) $$DIM=\frac{{A\;}_{intake}\;X\;B_{metal}\;X\;C_{factor}}{D_{weight}}\;$$

A_intake,_ B_metal_, C_factor_, and D_weight_ are the average daily intake of the oil samples by humans, metals present in the oil sample, a constant that is the conversion factor (0.085) and the average human weight (55.5 kg) respectively. A factor of 0.0527 kg person^−1^ day^−1^ was used as the average daily intake of palm oil per person per day [40].

### 2.6 Health Risk Index (HRI)

This was achieved by comparison of the ratio of the daily intake of metals in the oil to the oral reference dose (RfD) [40].

(3) $$HRI=\frac{DIM}{RfD}\;$$

The RfD is quantitatively used in risk assessment to evaluate the ingestion of a non-carcinogenic contaminant per milligram per kilogram per day. An HRI greater than one signifies that the population suffers a health risk when such food is consumed [2,39].

### 2.7 The Target Hazard Quotient (THQ)

It was of paramount importance to investigate the health risk associated with the consumption of the palm oil. This was achieved by the use of the Target Hazard Quotient (Eq. (4)). The THQ describes the ratio of the evaluated dose to the reference dose by the following equation [2,39,41].

(4) $$THQ=\frac{Efr\;X\;ED\;XFI\;XMC\;X\;0.001}{RfD\;XBW\;XAT}\;$$

The exposure frequency (*Efr*) was calculated as 365 days in a year and the duration (ED) as 60 years for an adult. FI and MC signify the ingestion and metal concentration in the food. The average body weight (BW) and exposure time (AT) are 60 kg and 365 days in 60 years for adults when considering non-carcinogenic effects. A THQ value greater than one is very risky to the population consuming such food [2,39].

## 3 Results and discussion

### 3.1 Soil properties

Soil properties such as pH, electrical conductivity, organic matter, and particle size are significant contributors to bioaccumulation and magnifications of metals in plant parts especially the fruits and bioaccumulation of metals in different fractions of the soil. The study on the soil properties and the bioavailability of the metals in the soil to investigated toxicity and mobility has been extensively discussed in Olafisoye et al., 2016 [42,43]. The soils investigated had a pH of four which shows acidic soils and hence an increase in toxicity and mobility of metals with decrease in pH, particle size and organic matter. In the present study toxic metals (Cd, Co, Cr, Ni) were found in soil and palm oil at elevated concentrations so were the essential metals (Cu, Fe, Mn, Zn) which invariably would be toxic at high concentrations. Metals such as Cu, Zn, Fe and Mg possesses antioxidant properties and are components of antioxidant enzymes. Acidic soils generally favour the transport of metals from soil to edible plant parts. This assessed the possible human health threats linked with the intake of palm oil contaminated with toxic metals. Contaminated palm oil with essential metals above the recommended values also become toxic. A possible route of human contact to slow poisoning is from the ingestion of contaminated food [42,43]. Several sources of contamination of the oil palm plantations were identified in this study such as anthropogenic sources of metal pollution in soil mostly from industries and unwholesome farming practices. Application of fertilizers, untreated sewage water from municipal and industrial applications, leaching from dumpsites, fertilizer applications, and waste from mining and crude oil spills have contributed significantly to the build-up of metals in soils. Ayodele and Oluyomi [9] reported on the issue of contamination arising during cultivation of the oil palm tree and harvesting of the oil palm fruit due to pre-planting and post-planting farming practices. The application and use of fungicides and fertilizers residues, palm oil production using corroded vessels and storage of the palm oil in metal vessels are great contributors to metal build-up in the oil [18,44].

### 3.2 Metal concentrations in soil and palm oil

The results for the analysis of metals in virgin palm oil and soil are presented in Tables 1 & 2 respectively. The results revealed detectable levels of the metals as most of the metals in palm oil and soils exceeded the WHO permissible limits for metals in vegetable oils and soil [45]. The authors observed that the metal concentration in the soils were a bit lower than those in the palm oil due to bioaccumulation and biomagnifications in the tissues of the plant. This is in agreement with the study by El-Hassain et al. [46] that cultivated plants in polluted soils would have higher levels of heavy metals, as well as exceeding the permissible levels.

**Table 1:** Concentration of metals in palm oil (mg/L) (n = 3).

Location	Cd	Co	Cr	Cu	Fe	Mn	Ni	Zn
Abak	12.7 ± 0.001	9.8 ± 0.003	5.6 ± 0.004	7.0 ± 0.002	21.1 ± 0.007	6.6 ± 0.001	6.8 ± 0.001	8.3 ± 0.002
Acharu	13.9 ± 0.001	10.4 ± 0.001	4.7 ± 0.001	11.7 ± 0.001	9.1 ± 0.004	7.3 ± 0.004	16.4 ± 0.003	34.2 ± 0.001
Agbarho	68.3 ± 0.001	23.9 ± 0.001	3.4 ± 0.002	7.5 ± 0.001	15.9 ± 0.005	2.3 ± 0.001	8.9 ± 0.005	12.6 ± 0.001
Ago-Emokpae	14.0 ± 0.001	10.4 ± 0.001	6.8 ± 0.001	7.0 ± 0.003	16.8 ± 0.013	6.4 ± 0.001	20.2 ± 0.004	18.2 ± 0.002
Apoje	26.6 ± 0.002	45.8 ± 0.001	77.8 ± 0.002	8.3 ± 0.002	15.2 ± 0.009	45.6 ± 0.002	78.6 ± 0.001	162.1 ± 0.073
Badagry	12.7 ± 0.001	9.1 ± 0.002	2.8 ± 0.003	18.4 ± 0.003	17.4 ± 0.043	7.1 ± 0.001	18.4 ± 0.003	9.6 ± 0.001
Benin city	55.7 ± 0.002	33.2 ± 0.001	66.1 ± 0.002	9.1 ± 0.002	13.9 ± 0.005	7.3 ± 0.001	76.0 ± 0.002	10.0 ± 0.001
Igede-Ekiti	76.2 ± 0.002	55.1 ± 0.002	43.0 ± 0.002	9.0 ± 0.001	13.7 ± 0.002	7.6 ± 0.001	17.7 ± 0.001	5.3 ± 0.003
Ikire	13.8 ± 0.001	10.3 ± 0.001	3.2 ± 0.001	10.9 ± 0.001	18.4 ± 0.005	7.0 ± 0.001	18.2 ± 0.002	9.2 ± 0.001
Iresaapa	13.9 ± 0.001	13.1 ± 0.001	4.6 ± 0.001	8.2 ± 0.002	13.6 ± 0.001	6.8 ± 0.001	16.7 ± 0.001	32.9 ± 0.001
Nsukka	61.1 ± 0.001	66.5 ± 0.004	33.4 ± 0.002	2.2 ± 0.001	3.3 ± 0.002	48.2 ± 0.001	67.2 ± 0.003	45.2 ± 0.001
Okitipupa	38.9 ± 0.002	31.1 ± 0.002	2.8 ± 0.001	33.2 ± 0.002	11.2 ± 0.001	67.2 ± 0.003	67.2 ± 0.003	11.1 ± 0.001
Onishere	44.5 ± 0.0050	88.7 ± 0.002	1.1 ± 0.001	44.6 ± 0.006	67.3 ± 0.003	11.3 ± 0.002	54.3 ± 0.003	66.3 ± 0.002
Ubiaja	13.7 ± 0.001	10.2 ± 0.001	99.8 ± 0.001	7.6 ± 0.005	18.7 ± 0.023	3.7 ± 0.002	17.4 ± 0.001	64.2 ± 0.001
Umuabi	13.1 ± 0.001	10.0 ± 0.001	4.0 ± 0.001	10.1 ± 0.007	17.0 ± 0.003	26.4 ± 0.003	17.8 ± 0.002	77.9 ± 0.001

FAO/WHO maximum permissible limits for Cd, Co, Cr, Cu, Fe, Mn, Ni and Zn are 9, 0.06, 0.1, 30, 48, 20, 300 and 60 mg/L respectively.

**Table 2:** Concentration of metals in soils (mg/kg) (n = 2).

Location	Cd	Co	Cr	Cu	Fe	Mn	Ni	Zn
Abak	1.09 ± 0.2	9.42 ± 0.3	109.56 ± 0.1	39.11 ± 0.2	30.99 ± 0.3	0.88 ± 0.1	40.66 ± 0.1	63.11 ± 0.1
Acharu	1.16 ± 0.1	2.17 ± 0.1	45.67 ± 0.1	12.92 ± 0.1	40.34 ± 0.1	19.22 ± 0.1	37.24 ± 0.2	89.33 ± 0.2
Agbarho	1.66 ± 0.1	3.98 ± 0.2	132.21 ± 0.1	45.06 ± 0.4	12.34 ± 0.1	0.23 ± 0.2	39.11 ± 0.1	23.01 ± 0.1
Ago-Emokpae	0.64 ± 0.2	2.07 ± 0.1	25.50 ± 0.3	35.08 ± 0.1	9.11 ± 0.2	9.42 ± 0.3	30.27 ± 0.1	92.48 ± 0.1
Apoje	1.28 ± 0.1	1.03 ± 0.1	99.92 ± 0.1	33.22 ± 0.1	6.63 ± 0.1	0.45 ± 0.1	35.67 ± 0.1	96.67 ± 0.1
Badagry	4.26 ± 0.2	2.61 ± 0.1	108.67 ± 0.1	69.11 ± 0.1	1.81 ± 0.2	1.33 ± 0.2	49.08 ± 0.1	53.82 ± 0.2
Benin city	1.33 ± 0.1	1.32 ± 0.2	78.11 ± 0.1	23.02 ± 0.2	2.02 ± 0.1	29.38 ± 0.1	32.35 ± 0.3	107.33 ± 0.2
Igede-Ekiti	0.39 ± 0.2	1.45 ± 0.1	56.45 ± 0.1	33.41 ± 0.1	0.05 ± 0.1	0.76 ± 0.1	39.46 ± 0.2	78.62 ± 0.2
Ikire	0.27 ± 0.1	1.05 ± 0.2	34.99 ± 0.2	22.63 ± 0.1	1.10 ± 0.1	1.49 ± 0.2	44.76 ± 0.2	94.42 ± 0.1
Iresaapa	1.32 ± 0.1	2.55 ± 0.1	111.08 ± 0.1	30.82 ± 0.2	1.03 ± 0.1	0.75 ± 0.1	45.34 ± 0.1	12.36 ± 0.1
Nsukka	1.12 ± 0.1	2.23 ± 0.1	124.42 ± 0.3	28.56 ± 0.1	1.01 ± 0.2	0.12 ± 01	49.22 ± 0.1	37.43 ± 0.1
Okitipupa	0.12 ± 0.1	2.61 ± 0.2	66.89 ± 0.1	33.78 ± 0.2	1.01 ± 0.1	0.99 ± 0.2	34.04 ± 0.1	60.28 ± 0.1
Onishere	0.37 ± 0.1	0.80 ± 0.1	111.11 ± 0.3	37.99 ± 0.1	2.02 ± 0.1	0.62 ± 0.1	50.62 ± 0.2	75.07 ± 0.1
Ubiaja	0.32 ± 0.1	2.45 ± 0.1	42.90 ± 0.2	30.22 ± 0.1	1.06 ± 0.2	1.51 ± 0.1	59.11 ± 0.1	91.64 ± 0.3
Umuabi	0.32 ± 0.1	1.14 ± 0.1	111.02 ± 0.1	25.52 ± 0.1	1.13 ± 0.1	0.18 ± 0.1	51.02 ± 0.1	90.88 ± 0.2

Chromium, Copper, Nickel, and Zinc recorded rather high concentrations of metals in soils on all the fifteen sampling plantations in the study. Iron recorded a high concentration in Acharu while manganese was high in Acharu and Benin City respectively. Highest value of metals in soils was recorded in Chromium in Agbarho plantation. This is evident in Table 2. Rich deposits of Iron were found in Acharu. Acharu is a local government in Kogi state near Itakpe, a famous Iron smelting company in Nigeria. The soils of the area are also rich in Iron Ore. Iron is a trace element necessary for proper functioning of the body and haemoglobin. Benin City is a haven for Bronzes and Brass ornaments, smelting of metals, and artwork. High values of Copper and Zinc were recorded in soils sampled from Benin City. These metals are components of antioxidant metals in trace concentrations. The rather high concentrations of these metals in soil are toxic and detrimental to humans when transferred to consumable parts of plants. The high concentration in soil is fatal. Elevated concentrations of these metals in soils and their detrimental effect on humans have been broadly discussed in a previous study [42].

Table 2 shows the accumulation of metals from soil to palm oil. Chromium and Copper generally recorded high concentrations of metals in all plantations under study. A high value of Cadmium in palm oil was also noted in Igede Ekiti and Cobalt in Apoje. Igede-Ekiti, Nsukka and Onishere plantations respectively. Chromium metal recorded high concentrations of the metals in palm oil in Apoje, Benin City, and Ubiaja.

Copper and Nickel and iron recorded high values in palm oil in Onishere. High values were also noted for Nickel in Apoje, Benin City, and Okitipupa in palm oil. High values of Manganese, and Zinc metals were noted in palm oil in Apoje, Nsukka and Okitipupa respectively.

The plantations which recorded the highest value of metal was Apoje with the highest concentration of Zinc metal

The data obtained was compared with Balkhair and Ashraf [2]. Nearly all the concentrations of the metallic elements exceeded the permissible limits in palm oil and soils analysed from the plantations. The deleterious effects of the metals in elevated concentrations and their beneficial outcomes have been discussed in a previous study [42]. In addition, the bioavailability fractions in which the metals exist in soil gave an insight into their mobility and toxicity as expatiated in the previous study [[47], [48], [49], [50], [51]].

Metallic elements have the ability to be translocated from soil to various parts of the plants. Invariably researchers have studied the oil palm tree extensively as an effective crop in remediation technology. It is a cheap choice to phytoremediation because it is low in maintenance and eco-friendly. Soils are cleaned up as the oil palm tree translocated metallic elements from soils to parts of the plant such as fruits and stems. This provides interesting tools to sites contaminated by toxic metals. Researchers have studied the accumulation of metallic elements in parts of the oil palm tree such as the stem and the root. Research is limited in area of the virgin palm oil, which is the oil palm fruit to decide if accumulation may be higher in such parts [[51], [52], [53], [54], [55], [56], [57], [58]].

### 3.3 Risk assessment

Toxic metals on-route to humans and the food chain, via consumption of food crops grown on contaminated soils [2,59]. This is especially hazardous and a critical health risk issue when the metallic elements exceed the permissible levels in palm oil. The food chain as a pathway of toxic metallic elements is vital in risk assessment in developing countries like Nigeria since ingestion of palm oil remains deregulated. Palm oil is a stable ingredient for the preparation of most dishes in Nigeria and consumed regularly by the population at large. Horiguchi and colleagues [60] implied that the intake of metals does not equate the concentrations absorbed by humans. He further explained that part of the absorbed dose is eliminated through various biochemical processes. The remainder portion becomes bio accumulated and biomagnified in humans. This study can be used to assess crop pollution and prospective risk assessment. There is a risk to the masses since they are not aware of the nutritional content of the palm oil consumed daily in the form of an ingredient of most of their stable food.

Table 3a, Table 3b, Table 3c present the values for the entire risk assessment factors such as the Accumulation Factor (AF), Daily Intake of Metals (DIM), Health Risk Assessment (HRI) and Target Hazard Quotient (THQ) calculated for each sampling location while Table 4 highlights the minimum and maximum values for each factor in the respective metals.

**Table 3a:** AF, DIM (mg/kg/person/day), HRI and THQ values for Cd, Co and Cr.

	Cd				Co				Cr			
	AF	DIM	HRI	THQ	AF	DIM	HRI	THQ	AF	DIM	HRI	THQ
Abak	0.12	0.012	11.77	0.2032	1.04	0.010	2.86	0.0480	0.05	0.004	0.03	6.38E^−4^
Acharu	5.85	0.008	7.84	0.2224	8.45	0.012	3.43	0.0450	0.12	0.004	0.03	5.36E^−4^
Agbarho	17.16	0.016	15.68	0.0368	14.4	0.052	14.86	0.1092	0.26	0.002	0.014	3.88E^−4^
Ago-Emokpae	5.02	0.016	15.68	0.1024	21.90	0.072	20.57	0.0480	0.67	0.006	0.04	7.75E^−4^
Apoje	20.78	0.08	78.43	0.0720	44.5	0.024	6.86	0.2093	0.78	0.06	0.43	8.87E^−3^
Badagry	2.98	0.012	11.77	0.1136	3.49	0.010	2.86	0.0416	0.03	0.002	0.014	3.19E^−4^
Benin City	41.90	0.08	78.43	0.1168	25.2	0.04	11.43	0.1517	0.85	0.06	0.43	7.54E^−3^
Igede-Ekiti	52.60	0.12	117.65	0.1216	141.3	0.06	17.14	0.2518	0.76	0.04	0.29	4.90E^−4^
Ikire	51.10	0.010	9.80	0.1120	9.81	0.010	2.86	0.0471	0.09	0.009	0.06	3.65E^−4^
Iresa-apa	10.50	0.24	235.29	0.1088	5.14	0.010	2.86	0.0600	0.04	0.006	0.04	5.24E^−4^
Nsukka	29.80	0.012	11.77	0.7712	54.6	0.04	11.43	0.3039	0.27	0.0026	0.02	3.81E^−4^
Okitipupa	324.20	0.075	78.43	1.0752	11.92	0.04	11.43	0.1422	0.04	0.026	0.19	3.19E^−4^
Onishere	120.30	0.078	78.43	0.1808	110.9	0.04	11.43	0.4054	0.009	0.0008	0.006	1.25E^−4^
Ubiaja	4.16	0.004	3.92	0.0592	42.8	0.0010	2.86	0.0466	2.23	0.08	0.57	1.10E^−2^
Umuabi	40.93	0.0010	0.98	0.4220	8.77	0.0010	2.86	0.0457	0.04	0.004	0.03	4.56E^−4^

**Table 3b:** AF, DIM (mg/kg^/^person^/^day), HRI and THQ values for Fe, Mn and Ni.

	Fe				Mn				Ni			
	AF	DIM	HRI	THQ	AF	DIM	HRI	THQ	AF	DIM	HRI	THQ
Abak	0.68	0.016	3.0	6.75E^−6^	7.5	0.006	0.04	7.52E^−4^	0.17	0.006	0.3	5.4E^−3^
Acharu	0.52	0.008	0.4	2.91E^−6^	0.34	0.006	0.04	8.32E^−4^	0.18	0.014	0.7	1.3E^−2^
Agbarho	1.29	0.012	0.6	5.09E^−6^	10	0.0018	0.01	2.62E^−4^	0.8	0.008	0.4	7.1E^−3^
Ago-Emokpae	1.84	0.014	0.7	5.38E^−6^	0.68	0.006	0.04	7.30E^−4^	0.66	0.016	0.8	1.6E^−3^
Apoje	2.29	0.012	0.6	4.86E^−6^	101.3	0.04	0.29	5.20E^−3^	2.20	0.006	0.3	6.3E^−2^
Badagry	9.61	0.014	0.7	5.87E^−6^	5.34	0.006	0.04	8.09E^−4^	0.38	0.014	0.7	1.5E^−2^
Benin City	6.89	0.012	0.6	4.45E^−6^	0.25	0.006	0.04	8.32E^−4^	2.35	0.06	3.0	6.1E^−2^
Igede-Ekiti	27.4	0.012	0.6	4.39E^−6^	10	0.006	0.04	8.66E^−4^	0.45	0.014	0.7	1.4E^−2^
Ikire	16.73	0.014	0.7	5.89E^−6^	4.70	0.006	0.04	7.98E^−4^	0.41	0.014	0.7	1.5E^−2^
Iresa-apa	13.20	0.002	0.1	4.35E^−6^	9.07	0.006	0.04	7.75E^−4^	0.37	0.014	0.7	1.3E^−2^
Nsukka	3.27	0.010	0.5	1.06E^−6^	1.7	0.038	0.29	5.50E^−3^	1.37	0.06	3.0	5.4E^−2^
Okitipupa	11.09	0.006	0.3	3.58E^−6^	67.9	0.006	0.04	7.66E^−3^	1.94	0.06	3.0	5.4E^−2^
Onishere	33.3	0.006	0.3	2.15E^−5^	18.2	0.010	0.07	1.29E^−3^	1.07	0.04	2.0	4.3E^−2^
Ubiaja	17.6	0.014	0.7	5.98E^−6^	2.45	0.004	0.03	4.22E^−4^	0.29	0.014	0.7	1.4E^−2^
Umuabi	15.04	0.014	0.7	5.44E^−6^	22.40	0.02	0.14	3.21E^−4^	0.34	0.008	0.4	1.4E^−2^

**Table 3c:** AF, DIM (mg/kg^/^person/day), HRI and THQ values for Zn and Cu.

	Zn				Cu			
	AF	DIM	HRI	THQ	AF	DIM	HRI	THQ
Abak	0.13	0.006	0.0045	9.96E^−5^	0.17	0.004	0.003	7.49E^−5^
Acharu	0.09	0.02	0.0150	4.11E^−4^	0.54	0.010	0.0007	1.25E^−4^
Agbarho	0.32	0.010	0.0075	1.51E^−4^	0.17	0.006	0.004	5.63E^−4^
Ago-Emokpae	0.20	0.014	0.0105	2.18E^−4^	0.20	0.004	0.003	7.49E^−5^
Apoje	1.68	0.12	0.0902	1.95E^−3^	0.25	0.009	0.006	8.88E^−5^
Badagry	0.18	0.008	0.0060	1.15E^−4^	0.27	0.014	0.009	1.97E^−4^
Benin City	0.09	0.008	0.0060	1.20E^−4^	0.40	0.008	0.005	9.74E^−4^
Igede-Ekiti	0.07	0.004	0.0030	6.36E^−5^	0.27	0.008	0.005	9.53E^−5^
Ikire	0.10	0.008	0.0060	1.11E^−4^	0.48	0.002	0.001	1.17E^−4^
Iresa-apa	2.66	0.02	0.0150	3.95E^−4^	0.27	0.006	0.004	8.77E^−5^
Nsukka	1.21	0.04	0.0300	5.43E^−4^	0.08	0.0016	0.001	2.35E^−5^
Okitipupa	0.18	0.006	0.0045	1.33E^−4^	0.98	0.02	0.01	3.55E^−4^
Onishere	0.88	0.06	0.045	7.96E^−4^	1.17	0.04	0.03	4.77E^−4^
Ubiaja	0.7	0.06	0.045	7.71E^−4^	0.25	0.006	0.004	8.13E^−4^
Umuabi	0.86	0.06	0.045	9.35E^−4^	0.40	0.08	0.05	1.08E^−3^

**Table 4:** AF, DIM, HRI and THQ maximum and minimum values in metals.

Metal	AF Highest Value	AF Lowest Value	DIM Highest Value	DIM Lowest Value	HRI Highest Value	HRI Lowest Value	THQ Highest Value	THQ Lowest Value
Ca	324.2 (Okitipupa)	0.12 (Abak)	0.24 (Iresa-Apa)	0.001 (Umuabi)	235.29 (Iresa-Apa)	0.98(Umuabi)	1.0752 (Okitipupa)	0.0368(Agbarho)
Co	141.3 (Igede-Ekiti)	1.04 (Abak)	0.072 (Ago-Emokpae)	0.001 (Ubiaja/Umuabi)	20.57 (Ago-Emokpae)	2.86 (Abak, Badagry, Ikire, Iresa-Apa, Ubiaja, Umuabi)	0.4054 (Onishere)	0.0416 (Badagry)
Fe	33.3 (Onishere)	0.52 (Acharu)	0.016 (Abak)	0.002 (Iresa-Apa)	3.0 (Abak)	0.1 (Iresa-Apa)	2.15E^−05^ (Onishere)	1.06E^−06^ (Nsukka)
Cr	2.23 (Ubiaja)	0.03 (Badagry)	2.23 (Ubiaja)	0.03 (Badagry)	0.57 (Ubiaja)	0.006 (Onishere)	1.10E^−02^ (Ubiaja)	1.25E^−04^ (Onishere)
Mn	101.3 (Apoje)	0.25 (Benin City)	0.04 (Apoje)	0.0018 (Agbarho)	0.29 (Apoje, Nsukka)	0.01 (Agbarho)	7.66E^−03^ (Okitipupa)	0.01 (Agbarho)
Ni	2.35 (Ubiaja)	0.29 (Ubiaja)	0.06 (Benin City, Nsukka, Okitipupa)	0.006 (Abak/Apoje)	3.0 (Benin City, Nsukka, Okitipupa)	0.3 (Abak/Apoje)	6.3E^−02^ (Apoje)	1.6E^−03^ (Agbarho)
Zn	2.66 (Iresa-Apa)	0.07 (Igede-Ekiti)	0.12 (Apoje)	0.004 (Igede-Ekiti)	0.0902 (Apoje)	0.003 (Igede-Ekiti)	1.95E^−03^ (Apoje)	6.36E^−05^ (Igede-Ekiti)
Cu	1.17 (Onishere)	0.17 (Abak/Agbarho)	0.08 (Umuabi)	0.0016 (Nsukka)	0.05 (Umuabi)	0.0007 (Acharu)	1.80E^−03^ (Umuabi)	2.35E^−05^ (Nsukka)

**Fig. 2:**
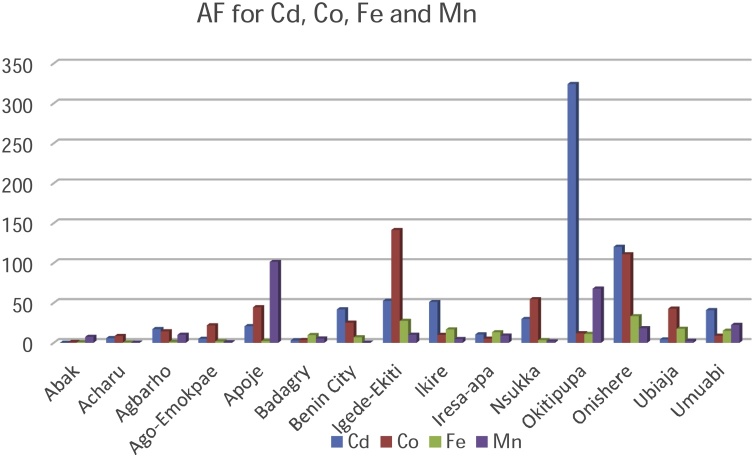
Accumulation Factor for Cadmium, Cobalt, Iron and Manganese.

**Fig. 3:**
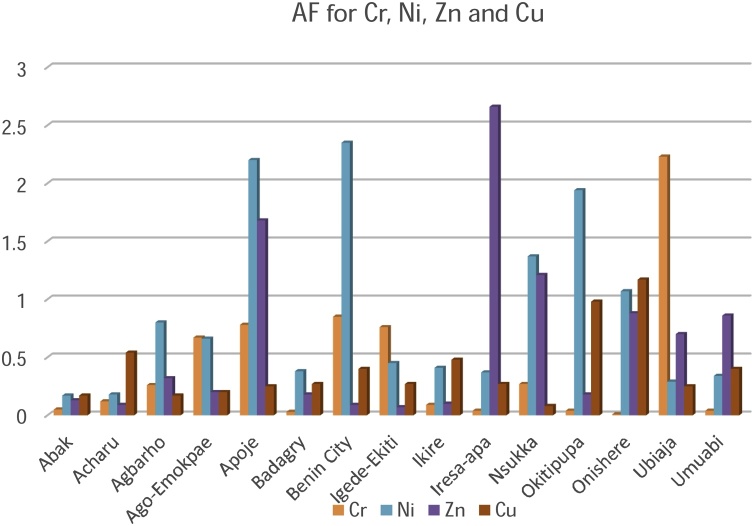
Accumulation Factor for Chromium, Nickel, Zinc and Copper.

The Accumulation Factor (AF) summarised in Table 4 and Figs. 2 & 3 can best be used to describe the degree in which the palm oil accumulates in the biological system, which is the oil palm fruit. The AF for the metallic elements in the palm oil ranged between 324.20-0.78 mg/kg and was greatest for Cd (324.20 mg/kg) in Okitipupa and Ni (141.3 mg/kg) in Igede-Ekiti plantations respectively. The AF value of 0.03 mg/kg (Cr) in Badagry plantation and 0.07 mg/kg (Zn) in Igede Ekiti plantations were the least AF values investigated respectively. The sequence of accumulation was can be described using the bioaccumulation factor, as Cd was the greatest bio accumulator in the palm oil.

The Accumulation factor (AF) for the metallic elements Cd, Co, Fe and Mn was greater than unity in all or most of the palm oil sampled from the plantations. This is an indication of bioaccumulation in the edible part of the oil palm tree, the fruit. AF factors lesser than one and very close to unity were recorded for metallic elements such as Ni, Cu, Cr and Zn. Lower AF values show low transfer of metallic elements and a lower risk of contamination.

Overall, the value of the Accumulation Factor is maximum for Cadmium in Okitipupa plantation and the lowest value was in Zinc (Igede Ekiti).

**Daily intake of metals and Health Risk Index**

**Fig. 4:**
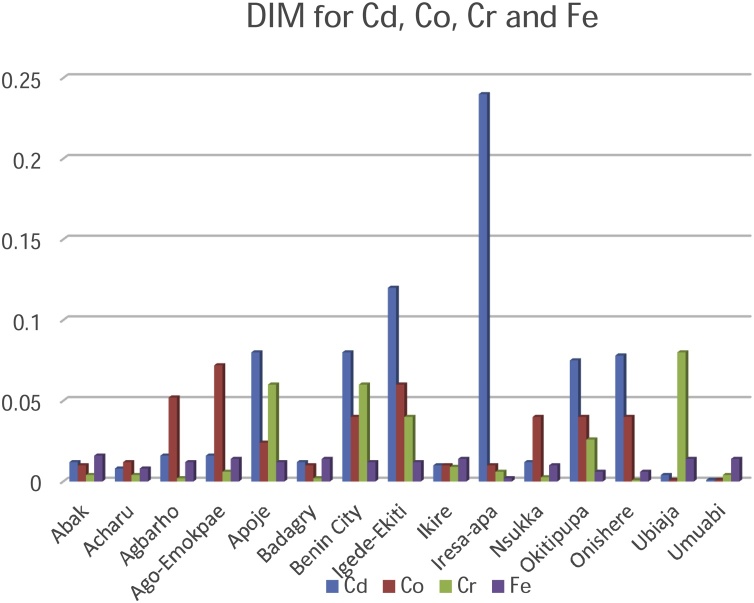
Daily Intake of Metals for Cadmium, Cobalt, Chromium and Iron.

**Fig. 5:**
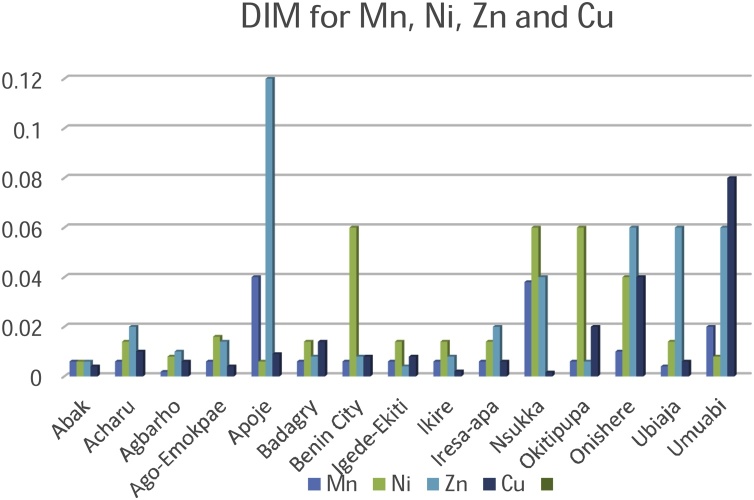
Daily Intake of Metals for Manganese, Nickel, Zinc and Copper.

Risk assessment of the palm oil in humans was best described by the Metals Daily Intake (DIM) and Health Risk Index (HRI) values. The exposure of the pollutants is the target organism. This is particularly risky and hazardous to the food chain. Risk assessment is of paramount significance in developing countries like Nigeria where the disposal of waste on agricultural lands is largely unregulated. Although several pathways of exposure such as the air, water, and soil may contribute to contamination, food is significantly a higher route of exposure. The consumption of palm oil as the stable ingredient in most foods is the pathway to the ingestion of the metallic elements in this study. The index that describes the average consumption of the palm oil are the DIM and HRI values (Table 3a, Table 3b, Table 3c). The values obtained for DIM and HRI in the plantations are summarised in Table 4 and Figs. 4 & 5 .

**Fig. 6:**
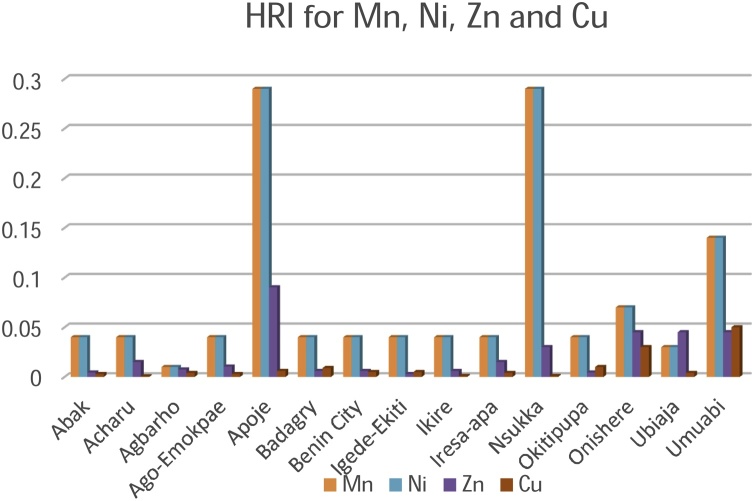
Health Risk Index for Manganese, Nickel, Zinc and Copper.

**Fig. 7:**
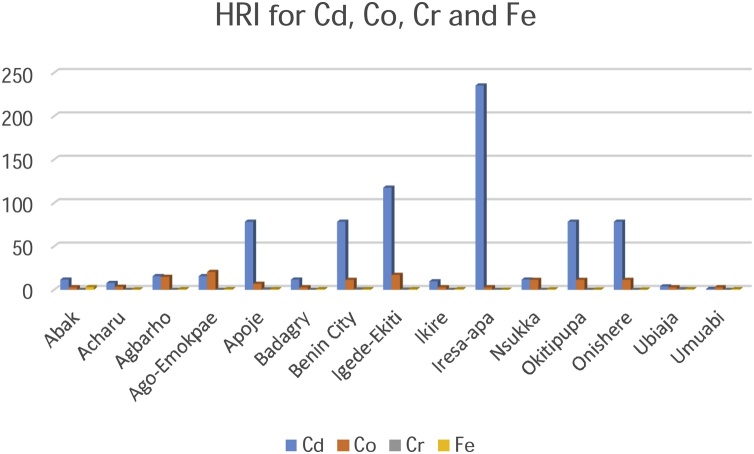
Health Risk Index for Cadmium, Cobalt, Chromium and Iron.

The DIM values of the contaminated palm oil were highest for Chromium (2.23) in Ubiaja plantation and Cadmium (0.24) in Iresa Apa plantation respectively. Lowest values were recorded for Chromium (0.0016) and Manganese (0.00180) at Nsukka and Agbarho plantations respectively. The DIM and HRI values were close to unity and greater than unity in most of the plantations indicating a high risk to the food chain. The Oral Reference Dose, (RfD) was earlier defined as a tool quantitatively used in risk assessment to evaluate the ingestion of a non-carcinogenic contaminant per milligram per kilogram per day. An HRI greater than one signifies that the population suffers a health risk when such food is consumed. The RfD is related to the HRI by an equation earlier explained [40]. Much lower values of HRI were found in metallic elements Cu, Mn and Zn. HRI values above unity signifies an indication of future toxicity to humans via the food chain. Essential and non-essential metallic elements at high and low concentration are detrimental at the risk assessment point of view. The values of HRI obtained in this study are in agreement with those obtained by [2,61,62] (Fig. 6, Fig. 7).

**Target Hazard Quotient**

**Fig. 8:**
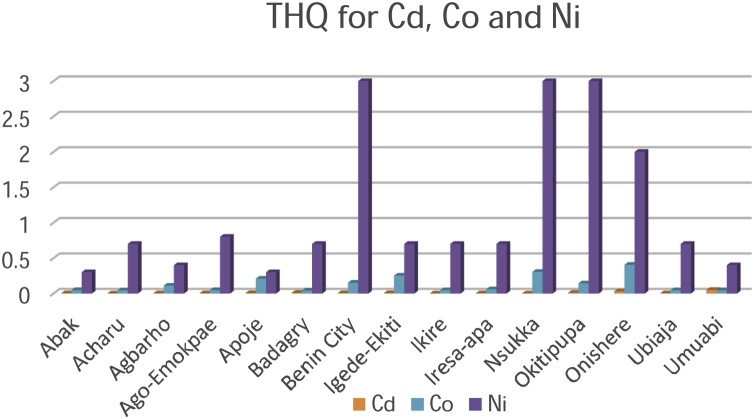
Target Hazard Quotient for Cadmium, Cobalt and Nickel.

**Fig. 9:**
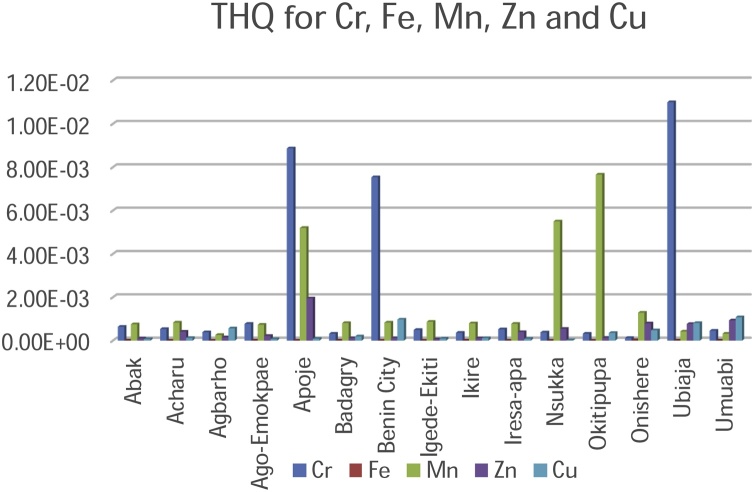
Target Hazard Quotient for Chromium, Iron, manganese, Zinc and Copper.

Another important tool for the evaluation of risk assessment with the intake of toxic metallic elements in food is the Target Hazard Quotient (THQ) [2,59]. Toxicity of the palm oil is a possibility since most of the THQ values obtained in the present study were above the permissible limits as shown in Table 4 and Figs. 8 & 9 . Although non-essential elements such as Cr, Ni and Cd were present in the palm oil at elevated concentrations, biochemical processes are available for their reduction in the human system. Biochemical processes emphasize that ingested metallic elements from food is not only absorbed into tissues as some may be excreted by the skin, kidneys etc.

#### 3.3.1 Correlation studies and the distribution of metals in palm oil and soils

**Fig. 10:**
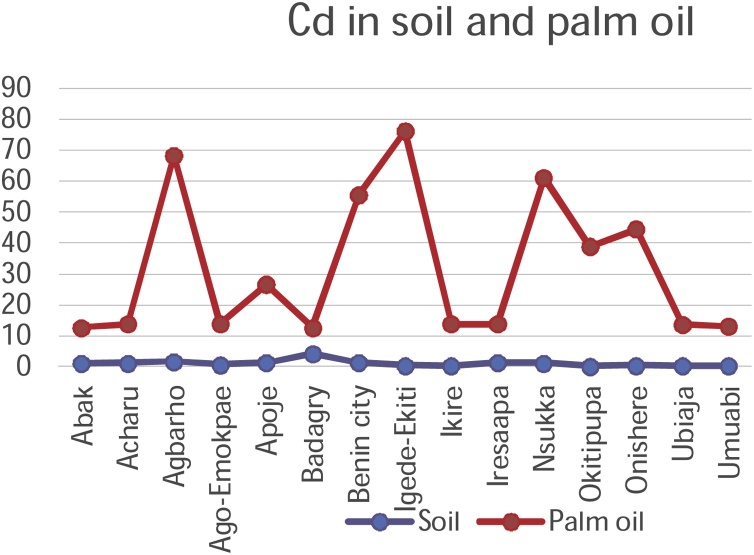
Correlation of Cadmium in soil and palm oil.

**Fig. 11:**
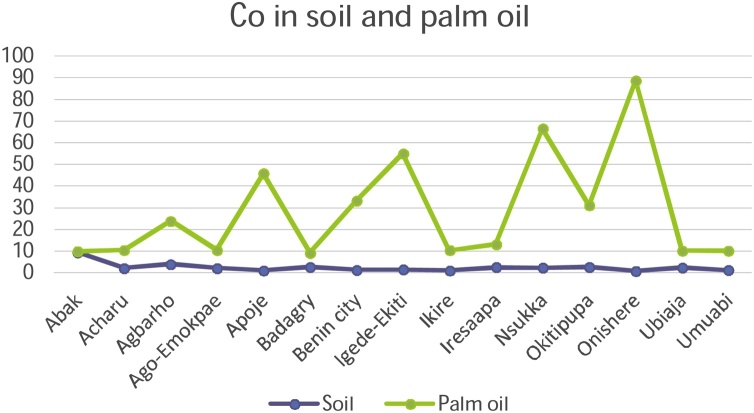
Correlation of Cobalt in soil and palm oil.

**Fig. 12:**
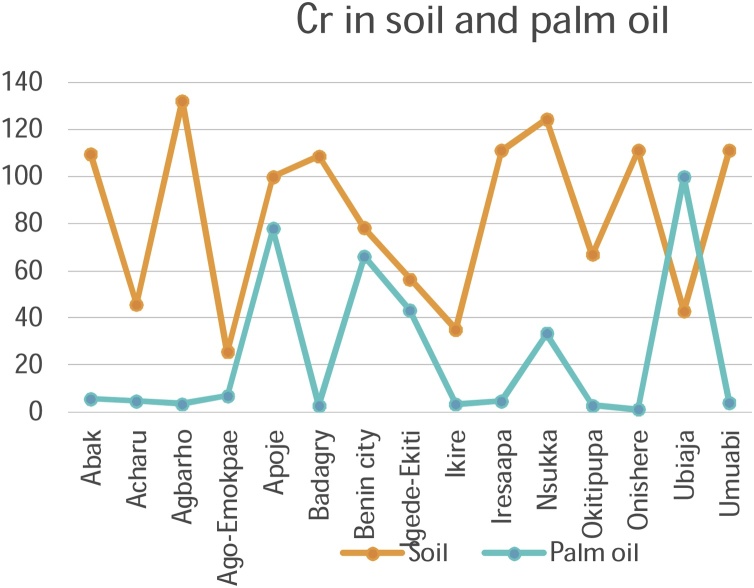
Correlation of Chromium in soil and palm oil.

**Fig. 13:**
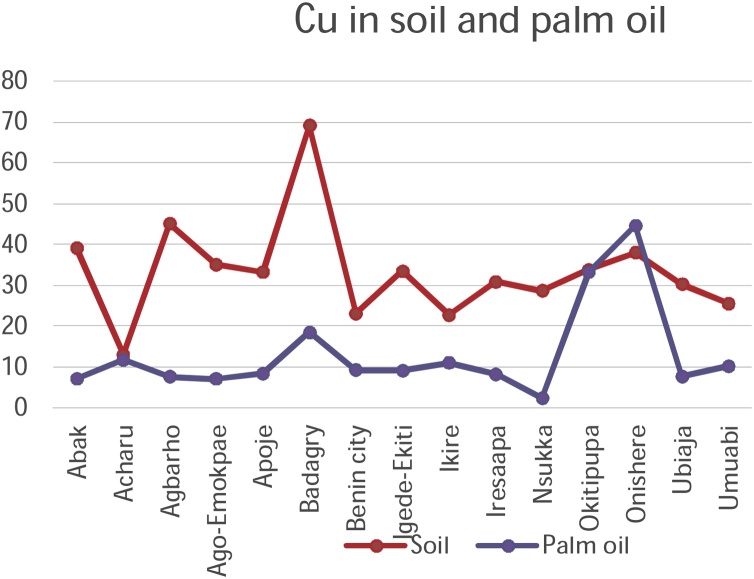
Correlation of Copper in soil and palm oil.

**Fig. 14:**
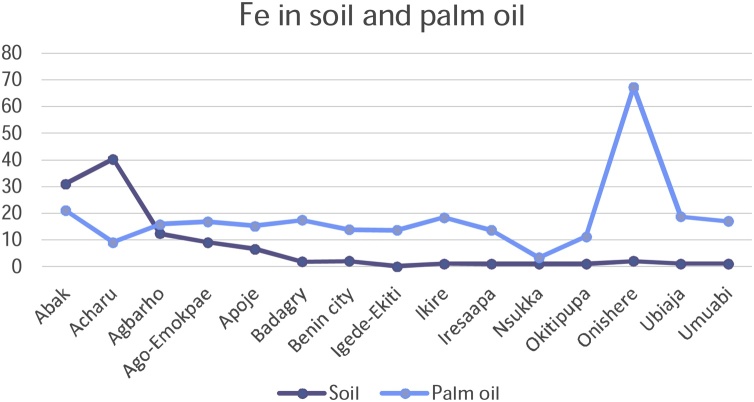
Correlation of Iron in soil and palm oil.

**Fig. 15:**
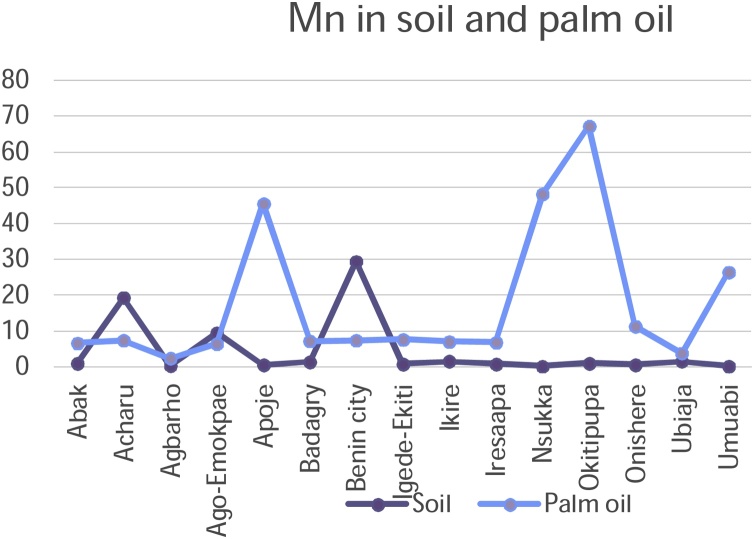
Correlation of Manganese in soil and palm oil.

**Fig. 16:**
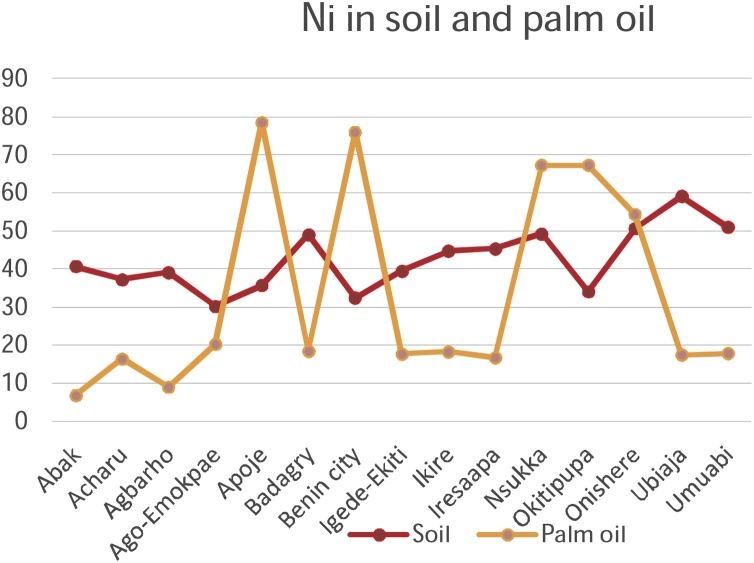
Correlation of Nickel in soil and palm oil.

**Fig. 17:**
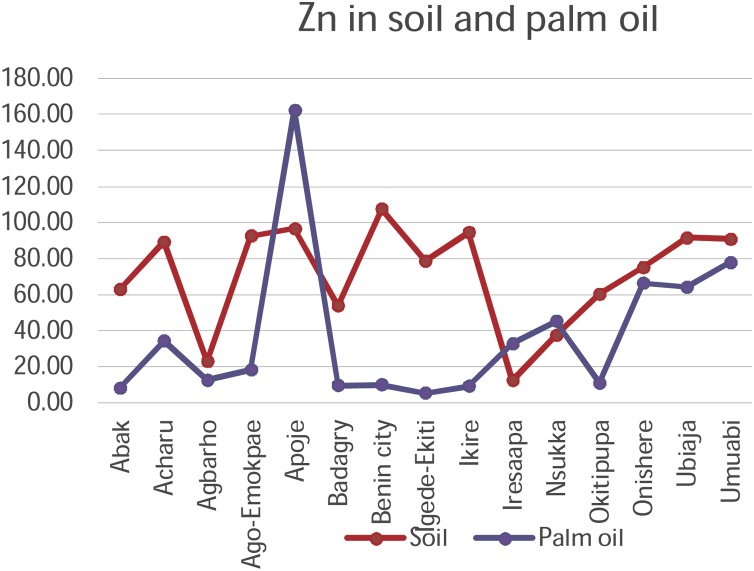
Correlation of Zinc in soil and palm oil.

The mobility and transfer of metals from soil to palm oil showed a positive correlation in the metals Copper and Zinc (+0.218 and +0.266) respectively. Negative correlation was exhibited with metals Cadmium, Cobalt, Chromium, Iron, Manganese and Nickel which showed negative correlation of -0.106, -0.352, -0.196, -0.113, -0.253 and -0.266 values respectively. Fig. 10, Fig. 11, Fig. 12, Fig. 13, Fig. 14, Fig. 15, Fig. 16, Fig. 17 depicts the correlation between the concentrations of metals in soil and palm oil.

## 4 Conclusions

Agronomical practices such as pesticides and fertilizers use has been extensively applied in agriculture due to the quest for improved yield. Agriculture produce has always been grown on acclaimed lands such as landfills and dumpsites. The unavailability of land for agricultural purposes, leached and drained soils and unsuitable topography has led to the quest for improved soil using sewage water, effluents, fertilizers and pesticides on agricultural lands. Pesticides, fertilizers, sewage water and effluents contain a relatively large concentration of metallic elements that are toxic to plants and humans when above the permissible limits. So do landfills and dumpsites. This present study assessed the concentration of palm oil pressed from oil palm fruits grown on anthropogenically contaminated oil palm plantation soils. This has led to an accumulation of metallic elements in the soil onto the palm oil. However, it was observed that the Accumulation Factor in the palm oil varied for each metal with Cadmium and Zinc being the top accumulators in palm oil. Hence, the palm oil sampled from most of the plantations was not desirable for human consumption but may be suitable for the agro allied and chemical industries and for the production of biodiesel. The plantation soils have a high concentration of metallic elements which bioaccumulated in the palm oil. The concentration of essential and non-essential metallic elements in elevated concentrations is toxic to human life and various signs and symptoms exhibit this.

To the best of the understanding of the authors, data is not available in the region for an effective risk assessment on the consumption of palm oil contaminated with metallic elements and its effect on humans. The present findings emphasize on anthropogenic contamination as the source of pollution in the palm oil. The intake of the palm oil has the potential of causing detrimental and undesirable health issues in the present and future. The results disclose high AF and HRI values above one and close to unity for most of the plantations. The investigation proffers a concise understanding on the prevailing situation and circumstances of palm oil poisoning and pollution with imminent and impending prospective health hazard assessment.

A crucial demand prevails to control the palm oil processed from the palm fruits in the region. In addition, policies and programmes must be put in place to forestall the accumulation of the metallic elements in the palm oil that will abate future health risks to the susceptible population of the region.

The study declares anthropogenic source of pollution as the source of point and non-point pollution in the palm oil. The values arrived at in this study will furnish baseline records and data for the consumption of palm oil in the region. Recommendation is required to prevent the onset of anthropogenic pollution into the farmlands and measures be taken for clean-up of soils before the embankment of pre and post planting operations.

## Declaration of Competing Interest

The authors declare no conflict of interest.
